# Supplementary material for: Computational Investigation of the pH Dependence of Stability of Melanosome Proteins: Implication for Melanosome formation and Disease
Source: Int J Mol Sci. 2021 Jul 31;22(15):8273. doi: 10.3390/ijms22158273 (PMC8347052; doi:10.3390/ijms22158273)
Supplement: Supplementary file 1 [file ijms-22-08273-s001.zip › ijms-1311257-supplementary.pdf]

**Supplementary Material**

# On the pH-dependence of stability of melanosome proteins: Implication for melanosome formation and disease

Mahesh Koirala[1, †], H.B. Mihiri Shashikala[1, †], Jacob Jeffries,[1] Bohua Wu[1], Stacie K. Loftus[3], Jonathan H. Zippin[2], Emil Alexov[1, *]

[1]Department of Physics, Clemson University, Clemson, SC 29634

[2]Department of Dermatology, Weill Cornell Medical College, New York, NY 10021

[3]Genetic Disease Research Branch, National Human Genome Research Branch, National Institutes of Health, Bethesda, MD 22066

* Correspondence: ealexov@clemson.edu

[†]Authors equally contributed to this work

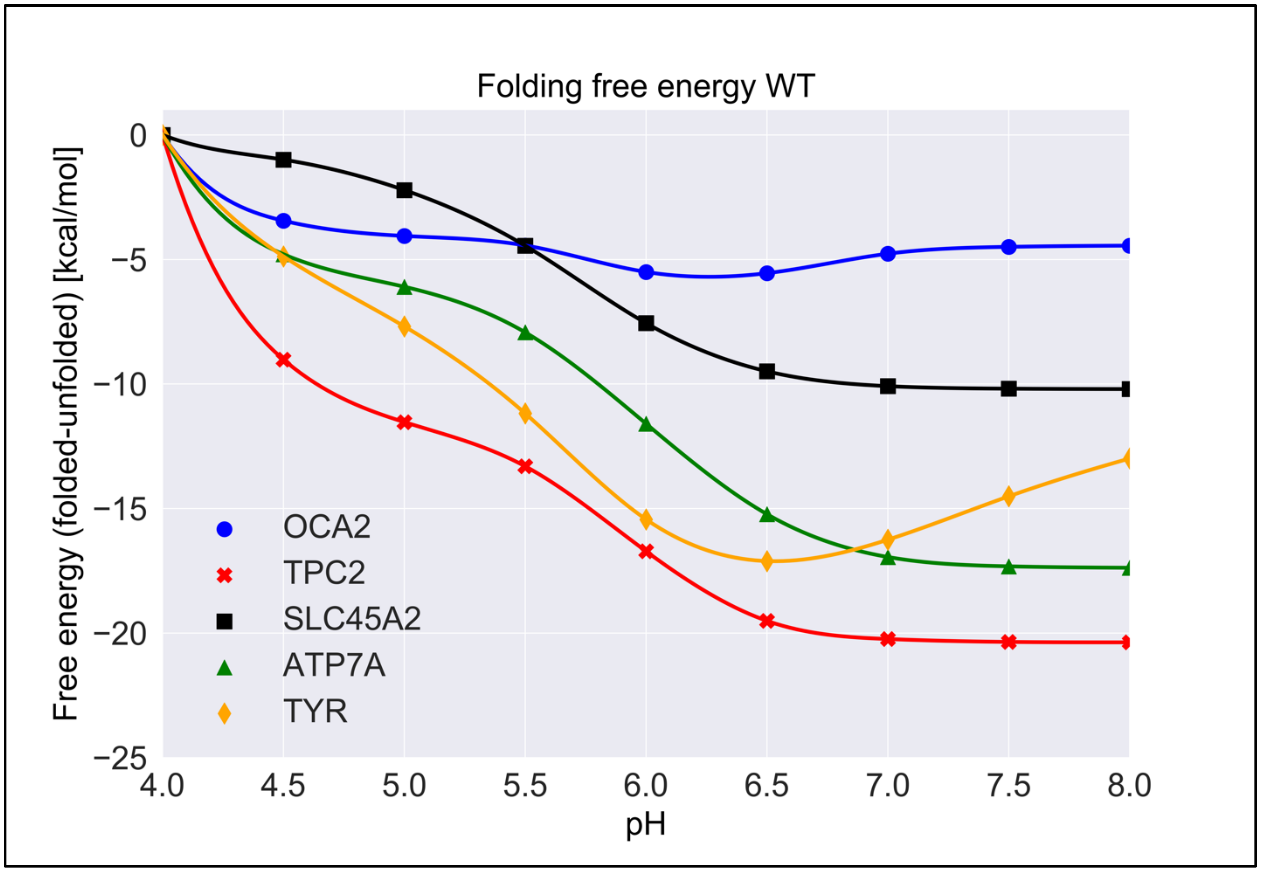


**Figure S1.** The pH-dependence of the folding free energy of wild type proteins from 20 MD snap shots within pH range 4-8.

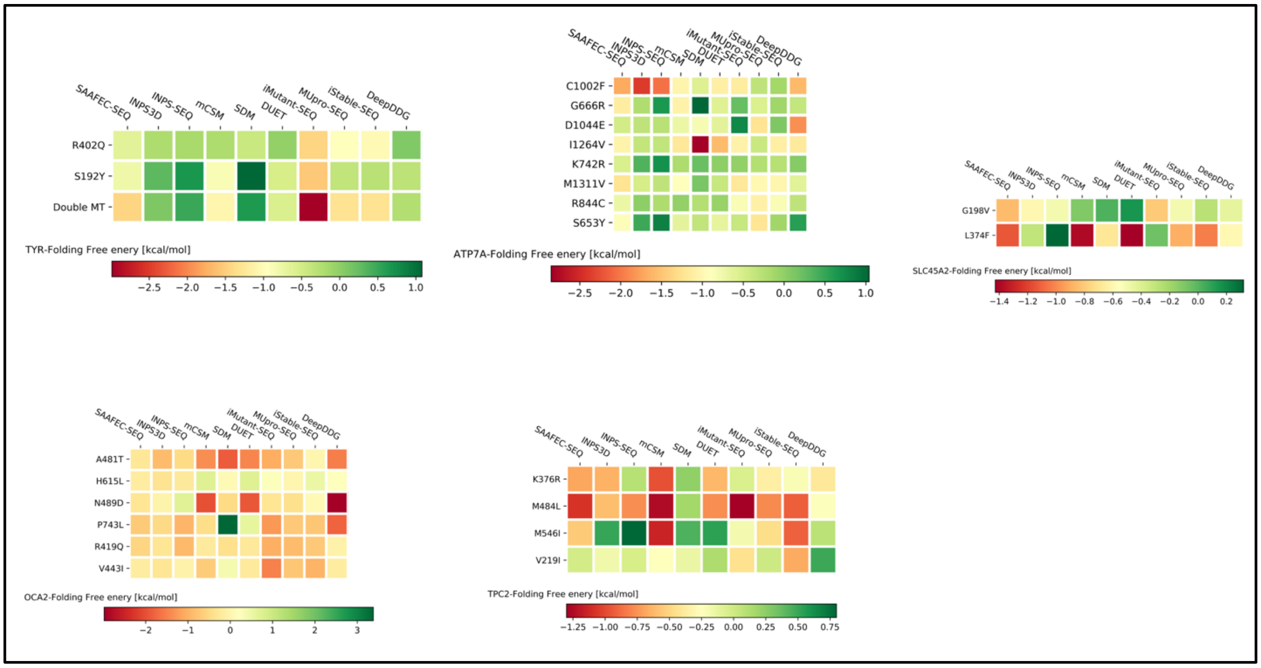


**Fig S2**. Effect of variants on protein stability computed from different tools, SAAFEC-SEQ INPS3D, INPS-SEQ, mCSM, SDM, DUET, I-Mutant-SEQ, MUpro-SEQ, iStable-SEQ and DeepDDG. Positive sign indicates stabilization, and the negative sign indicates destabilization[1-9].

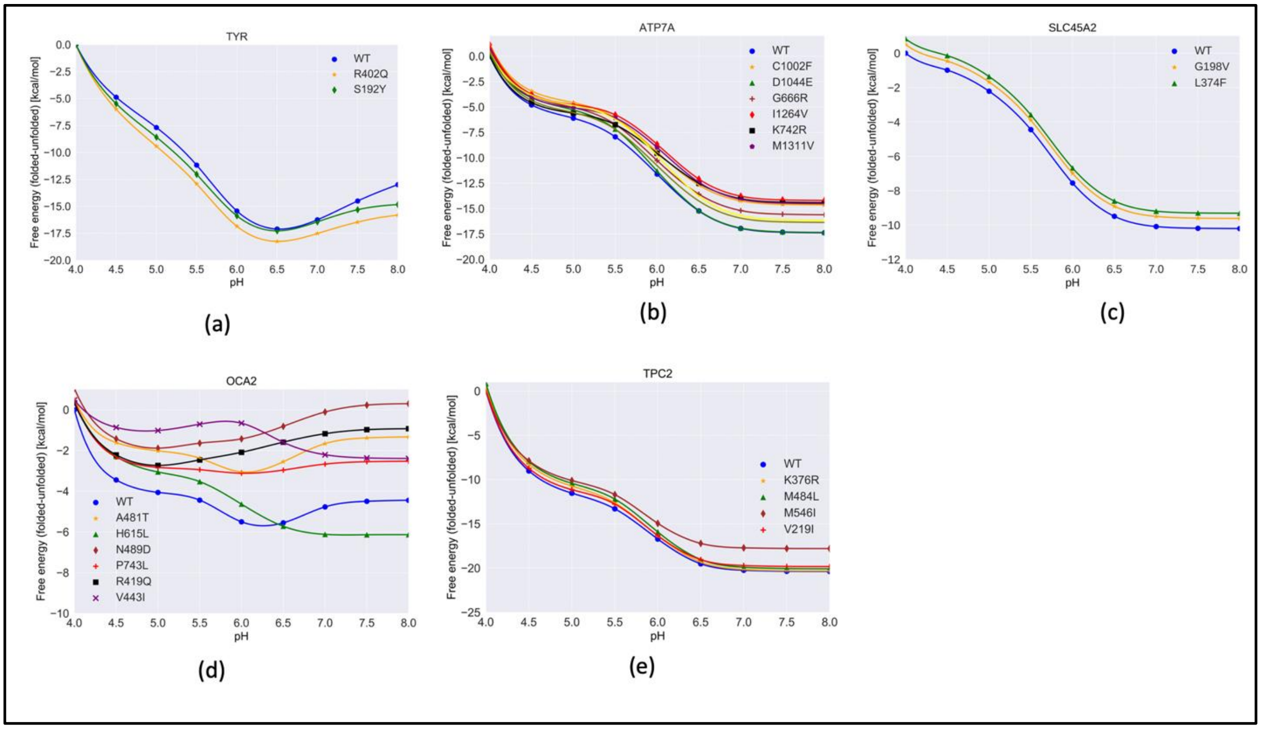


**Figure S3.** The pH-dependence of the folding free energy of wild type proteins and their mutants from 20 MD snapshots within pH range 4-8.

## Structures

### 3D model of TYR

The 3D structure of TYR is modeled from SWISS-MODE[10] from amino acid sequence of length (529 aa) taken from UniProt (ID: P14679) [11]. A template (PDB ID: 5M8P)[12] with percentage identity of 44 percent and covering 81 percent (19-452) of total sequence of TYR is selected to model the structure (Fig. S4). It can be seen that the structure contains two copper ions surrounded by Histidine.

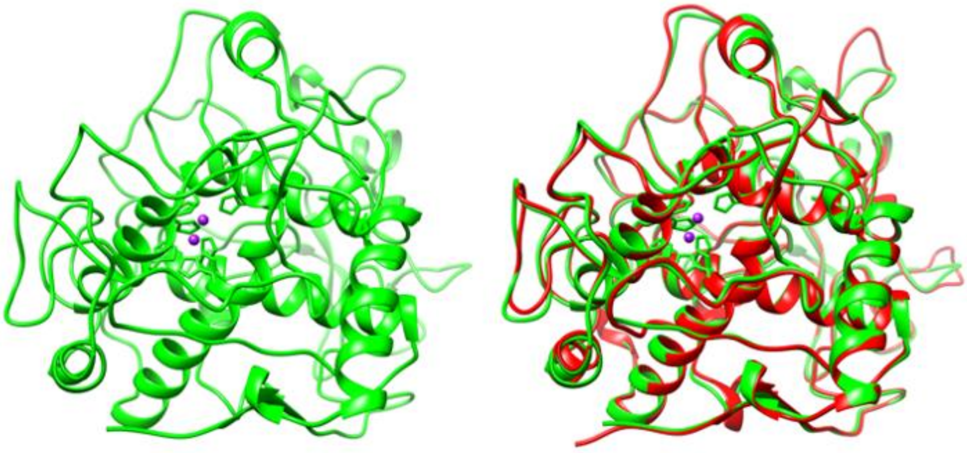

**Fig S4.** 3D model of TYR: (left) 3D structure of TYR; (right) superimposition of the TYR model (green) with its template (red)

**3D model of OCA2**

The 3D structure of OCA2 is modeled using Phyre2 [13] from the amino acid sequence of full length (838 aa). The sequence of OCA2 is taken from UniProt (ID: Q04671) [11]. A template (PDB ID: 4F35)[14] is selected with a percentage identity of 20 percent to query and covering 60 percent of the sequence of OCA2 (Fig. S5). It can be seen that the helical content is well preserved between the template and the model.

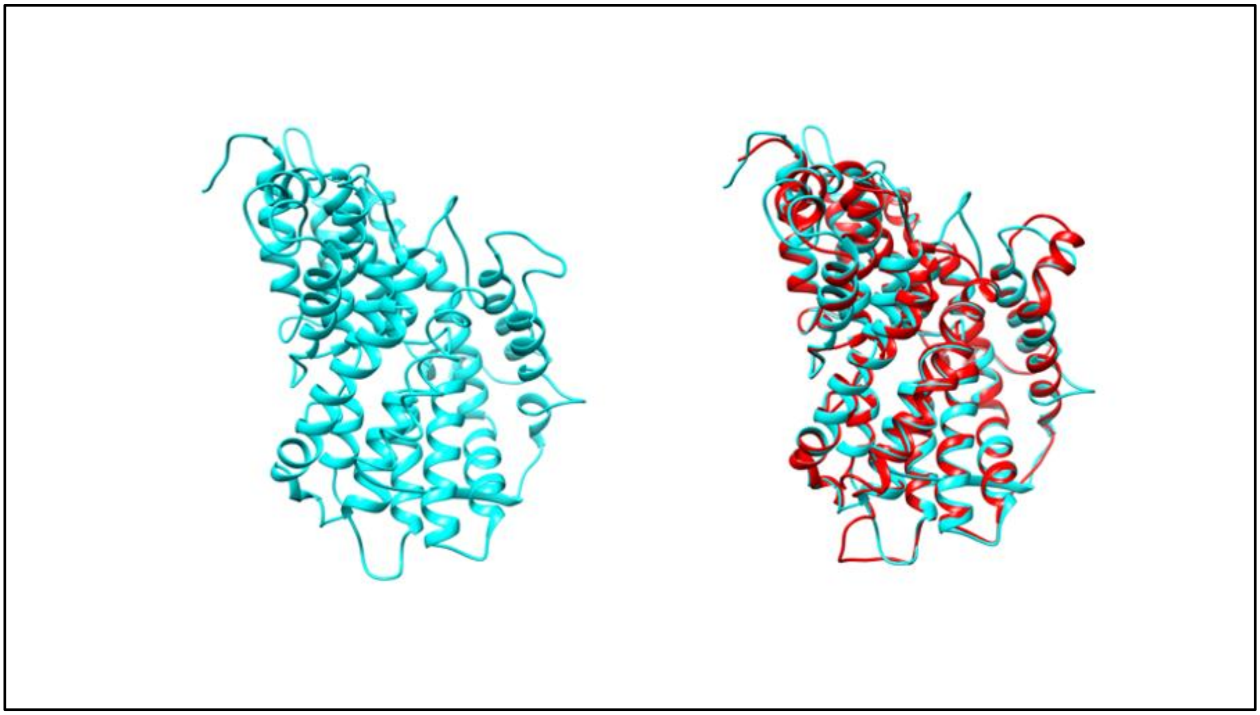

**Fig S5.** 3D model of OCA2: (left) 3D structure of OCA2; (right) superimposition of the OCA2 model (cyan) with its template (red)

**3D model of TPC2**

Crystal structure for TPC2 is available (PDB ID: 6NQ2)[15] and is a homodimer with 752 residues (Fig. S6)

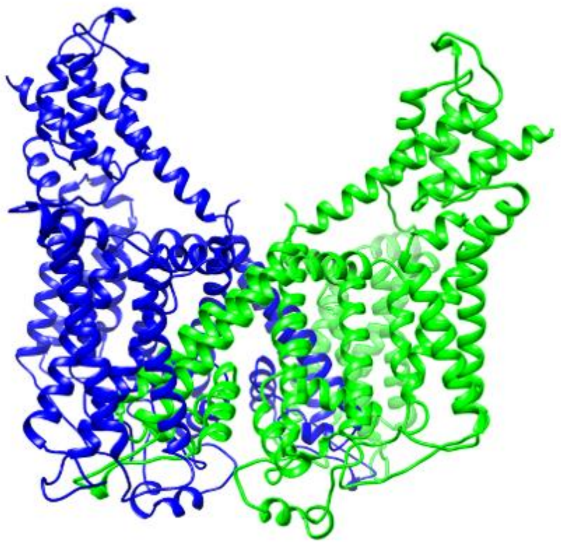

**Fig S6.** 3D model of TPC2: (blue) monomer A; (green) monomer B

**3D model of SLC45A2**

The 3D structure of SLC45A2 is modeled using Phyre2[13] . The sequence for it is taken from UniProt (ID: Q9UMX9)[11] with sequence length of 530 amino acids. A template chosen (PDB ID: 4YBQ) [16] covers 94 percent of the sequence with percentage identity of 14 percent (Fig. S7).

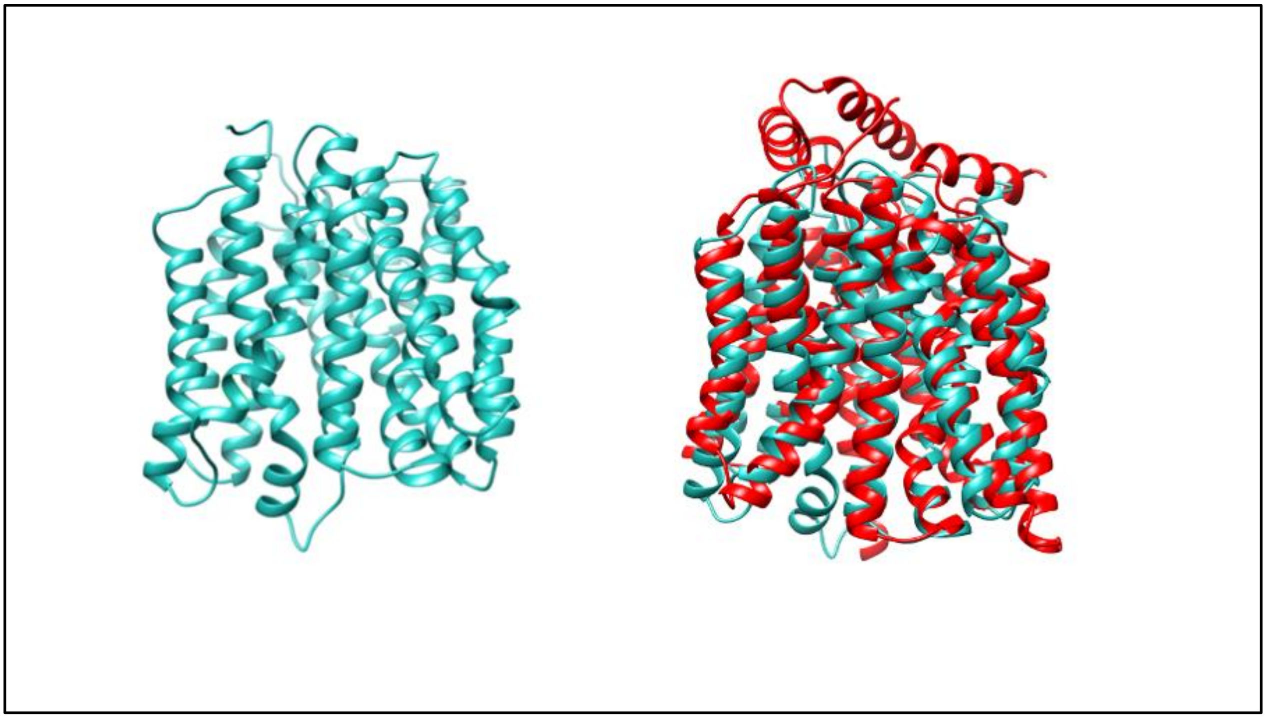

**Fig S7.** 3D model of SLC45A2: (left) 3D structure of SLC45A2; (right) superimposition of the SLC45A2 model (cyan) with its template (red)

**3D model of ATP7A**

The 3D modelling of this protein is also done with Phyre2[13]. The sequence is taken from UniProt (ID: Q04656)[11] with sequence length of 1500 amino acids. A template (PDB ID: 3RFU) [17] covers 57 percent of the sequence (646-1411) with percentage identity of 41 percent (Fig. S8).

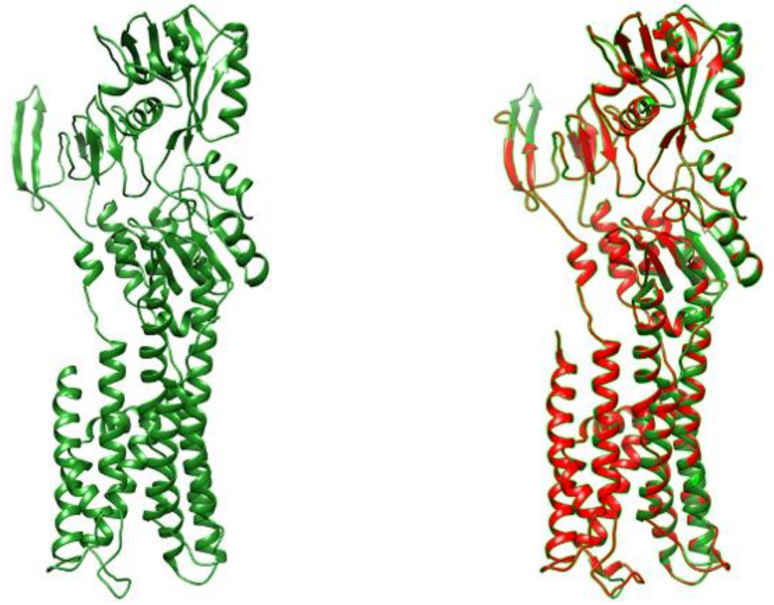

**Fig S8.** 3D model of ATP7A: (left) 3D structure of ATP7A; (right) superimposition of the ATP7A model (green) with its template (red)

**Sequence alignments:**

Sequence alignment of all the models with its template using T-Coffee web server [18] are shown below. An asterisk (*) indicates positions which have a single, fully conserved residue; a colon (:) indicates conservation between groups of strongly similar properties; a period (.) indicates conservation between group of weakly similar properties.

```
Model.pdb       HFPRACVSSKNLMEKECCPPWSGDRS----PCGQLSGRGSCQNILLSNAP
Template.pdb    QFPRQCATVEALRSGMCCPDLSPVSGPGTDRCGSSSGRGRCEAVTADSRP
                :*** *.: : * .  ***  *   .     **. **** *: :  .. *

Model.pdb       LGPQFPFTGVDDRESWPSVFYNRTCQCSGNFMGFNCGNCKFGFWGPNCTE
Template.pdb    HSPQYPHDGRDDREVWPLRFFNRTCHCNGNFSGHNCGTCRPGWRGAACDQ
                 .**:*. * **** **  *:****:*.*** *.***.*: *: *. * :

Model.pdb       RRLLVRRNIFDLSAPEKDKFFAYLTLAKHTISSDYVIPIGTYGQM--KNG
Template.pdb    RVLIVRRNLLDLSKEEKNHFVRALDMAKRTTHPLFVIATRRSEEILGPDG
                * *:****::***  **::*.  * :**:*  . :**.     ::   :*

Model.pdb       STPMFNDINIYDLFVWMHYYVSMDALLG-GSEIWRDIDFAHEAPAFLPWH
Template.pdb    NTPQFENISIYNYFVWTHYYSVKKTFLGVGQESFGEVDFSHEGPAFLTWH
                .** *::*.**: *** ***   .::** *.* : ::**:**.****.**

Model.pdb       RLFLLRWEQEIQKLTGDENFTIPYWDWRDA-EKCDICTDEYMGGQHPTNP
Template.pdb    RYHLLRLEKDMQEMLQEPSFSLPYWNFATGKNVCDICTDDLMGSRSNFDS
                * .*** *:::*::  : .*::***::  . : ******: **.:   :.

Model.pdb       NLLSPASFFSSWQIVCSRLEEYNSHQSLCNGTPEGPLRRNP-GNHDKSRT
Template.pdb    TLISPNSVFSQWRVVCDSLEDYDTLGTLCNSTEDGPIRRNPAGNVARPMV
                .*:** *.**.*::**. **:*::  :***.* :**:**** **  :. .

Model.pdb       PRLPSSADVEFCLSLTQYESGSMDKAANFSFRNTLEGFASPLTGIADASQ
Template.pdb    QRLPEPQDVAQCLEVGLFDTPPFYSNSTNSFRNTVEGFSDP-TGKYDPAV
                 ***.. **  **.:  ::: .: . :. *****:***:.* **  *.:

Model.pdb       SSMHNALHIYMNGTMSQVQGSANDPIFLLHHAFVDSIFEQWLRRHRPLQE
Template.pdb    SSLHNLAHLFLNGTGGQVHLSPNDPIFVLLHTFTDAVFDEWLRRYNADIS
                **:**  *:::*** .**: *.*****:* *:*.*::*::****:..  .

Model.pdb       VYPEANAPIGHNRESYMVPFIPLYRNGDFFISSKD-LGYDY------
Template.pdb    TFPLENAPIGHNRQYNMVPFWPPVTNTEMFVTAPDNLGYTYEIQWPS
                .:*  ********:  **** *   * ::*::: * *** *
```

**Fig S9**: Sequence alignment of TYR with percentage identity of 44 percent with its template, E-value=0 and Score=920.38

```
Model.pdb         R-G------------------SVETQVTIATAILAGVYALIIFEIVXRTL
Template.pdb      HRNSLIVLADVALFLALYHFLPFEHNVVLGISMLAFIAVLWLTEALHVTV
                  : .                  ..* :*.:. ::** : .* : * :  *:

Model.pdb         AAMLGSLAALAALAVIGDRPSLTXVVEWIDFETLALLFGMMILVAIFSET
Template.pdb      TAILVPVMAV-FFGIFETQAALNNFANSIIF----LFLGGFALAAAMHHQ
                  :*:* .: *:  :.::  :.:*. ..: * *    *::* : *.* : .

Model.pdb         GFFDYCAVKAYRLSRGRVWAMIIMLCLIAAVLSAFLDNVTTMLLFTPVTI
Template.pdb      GLDKVIADKVLAMAQGKMSVAVFMLFGVTALLSMWISNTATAAMMLPLVL
                  *: .  * *.  :::*:: . ::**  ::*:** ::.*.:*  :: *:.:

Model.pdb         RLCEVLNLDPRQVL-----IAEVIFTNIGGAATAIGDPPNVIIVSNQELR
Template.pdb      GVLSKVDADKQRSTYVFVLLGVAYSASIGGIATLVGSPPNAIAAAEVGL-
                   : . :: * ::       :. .  :.*** ** :*.***.* .::  *

Model.pdb         KMGLDFAGFTAXMFIGICLVLLVCFPLLRLLYWNRKLYNKEPSEIVELKX
Template.pdb      -------SFTDWMKFGLPTAMMMLPMAIAILYFL--LVNWDKGKV-----
                         .**  * :*:  .:::    : :**:   * * : .::

Model.pdb         EIXVSDGILLAKCLTVLGFVIFMFFLNSFVP---GIXLDLGWIAILGAIW
Template.pdb      -----------VTLGIFGLTVFLWIFSSPINAALGGFKSFDTLVALGAIL
                               * ::*:.:*::::.* :    *   .:. :. ****

Model.pdb         LLILADIXDFEIILXRVEWATLLFFAALFVLMEALAXLXLIEYVGEQTAL
Template.pdb      MLSFARVVHWKEIQKTADWGVLLLFGGGLCLSNVLKQTGTSVFLANA---
                  :* :* : .:: *   .:*..**:*.. : * :.*       ::.:

Model.pdb         LIKMVPEEQRLIAAIVLVVWVSALASSLIDNIPFTATMIPVLLNLSXDPE
Template.pdb      LSDMVSHMGIFVVILVVATFV-VFLTEFASNTASAALLIPVFATVAEA--
                  * .**..   ::. :*:..:* .: :.: .* . :* :***: .::

Model.pdb         VGLPAPPLMY-ALAFGA-CLGGNGTLIGASANVVCAGIAEQXGYGFSFME
Template.pdb      FGMS-PVLLSVLIAVAASCAFMLPVATPPNAIVFASGHIK-------QSE
                  .*:. * *:   :*..* *     .   ..* *..:*  :         *

Model.pdb         FFRLGFPMMVVSCTVGMCYLLVAXVVVGW-
Template.pdb      MMRVGLYLNI-AC----IGLLTAIAMLFWQ
                  ::*:*: : : :*      **.* .:: *
```

**Fig S10:** Sequence alignment of ATP7A with percentage identity of 47 percent with its template, E-value=0 and Score=1181.05

```
Model.pdb       R-G------------------SVETQVTIATAILAGVYALIIFEIVXRTL
Template.pdb    HRNSLIVLADVALFLALYHFLPFEHNVVLGISMLAFIAVLWLTEALHVTV
                : .                  ..* :*.:. ::** : .* : * :  *:

Model.pdb       AAMLGSLAALAALAVIGDRPSLTXVVEWIDFETLALLFGMMILVAIFSET
Template.pdb    TAILVPVMAV-FFGIFETQAALNNFANSIIF----LFLGGFALAAAMHHQ
                :*:* .: *:  :.::  :.:*. ..: * *    *::* : *.* : .

Model.pdb       GFFDYCAVKAYRLSRGRVWAMIIMLCLIAAVLSAFLDNVTTMLLFTPVTI
Template.pdb    GLDKVIADKVLAMAQGKMSVAVFMLFGVTALLSMWISNTATAAMMLPLVL
                *: .  * *.  :::*:: . ::**  ::*:** ::.*.:*  :: *:.:

Model.pdb       RLCEVLNLDPRQVL-----IAEVIFTNIGGAATAIGDPPNVIIVSNQELR
Template.pdb    GVLSKVDADKQRSTYVFVLLGVAYSASIGGIATLVGSPPNAIAAAEVGL-
                 : . :: * ::       :. .  :.*** ** :*.***.* .::  *

Model.pdb       KMGLDFAGFTAXMFIGICLVLLVCFPLLRLLYWNRKLYNKEPSEIVELKX
Template.pdb    -------SFTDWMKFGLPTAMMMLPMAIAILYFL--LVNWDKGKV-----
                       .**  * :*:  .:::    : :**:   * * : .::

Model.pdb       EIXVSDGILLAKCLTVLGFVIFMFFLNSFVP---GIXLDLGWIAILGAIW
Template.pdb    -----------VTLGIFGLTVFLWIFSSPINAALGGFKSFDTLVALGAIL
                             * ::*:.:*::::.* :    *   .:. :. ****

Model.pdb       LLILADIXDFEIILXRVEWATLLFFAALFVLMEALAXLXLIEYVGEQTAL
Template.pdb    MLSFARVVHWKEIQKTADWGVLLLFGGGLCLSNVLKQTGTSVFLANA---
                :* :* : .:: *   .:*..**:*.. : * :.*       ::.:

Model.pdb       LIKMVPEEQRLIAAIVLVVWVSALASSLIDNIPFTATMIPVLLNLSXDPE
Template.pdb    LSDMVSHMGIFVVILVVATFV-VFLTEFASNTASAALLIPVFATVAEA--
                * .**..   ::. :*:..:* .: :.: .* . :* :***: .::

Model.pdb       VGLPAPPLMY-ALAFGA-CLGGNGTLIGASANVVCAGIAEQXGYGFSFME
Template.pdb    FGMS-PVLLSVLIAVAASCAFMLPVATPPNAIVFASGHIK-------QSE
                .*:. * *:   :*..* *     .   ..* *..:*  :         *

Model.pdb       FFRLGFPMMVVSCTVGMCYLLVAXVVVGW-
Template.pdb    MMRVGLYLNI-AC----IGLLTAIAMLFWQ
                ::*:*: : : :*      **.* .:: *
```

**Fig S11:** Sequence alignment of OCA2 with percentage identity of 20 percent with its template, E-value=0 and Score=388.81

```
Model.pdb         -----------------EPPKRPTSRLIMXSMAM----------------
Template.pdb      MGWGGGGGCTPRPPIHQQPPERRVVTVVFLGLLLDLLAFTLLLPLLPGLL
                                   :**:* .  ::: .: :

Model.pdb         --------------------FGREFCYAVEAAYVTPVLLSVGLPSSLYSI
Template.pdb      ESHGRAHDPLYGSWQGGVDWFATAIGMPVEKRYNS--VLFGGLIGSAFS-
                                      *.  :  .**  * :  :*  ** .* :*

Model.pdb         VWFLSPILGFLLQPVVGSASDXCRSRWGRRRPYILTLGVMMLVGMALYLN
Template.pdb      ------VLQFLCAPLTGATSDCLGR-----RPVMLL------CLMGVA-T
                        :* **  *:.*::**         ** :*         *.:  .

Model.pdb         GATVVATMIGVVLFDFAADFIDGP-------IKAYLFDVCSXQDKEKGLX
Template.pdb      SYAVWATSRSFAAFL-ASRLIGGISKGNVSLSTAIVADLGSPLARSQGMA
                  . :* **  ... *  *: :*.*         .* : *: *   :.:*:

Model.pdb         YXALFTGFGGALGYLLGAIDWAXLELGRLLGTEFQVMFFFSALVLTLCFT
Template.pdb      VIGVAFSLGFTLGPMLGAS--LPLE----MAPWFALLFAASDLLFIFCFL
                    .:  .:* :** :***     **    :.. * ::*  * *:: :**

Model.pdb         VXLCSIS---------------------EAPLTEVAKGIPPQQTPQDPPL
Template.pdb      PETLPLEKRAPSIALGFRDAADLLSPLALLRFSAVARG-------QDPP-
                      .:.                        :: **:*       ****

Model.pdb         SSDGMYEYGSIEKVKNGYVNPXYRYLCISXLIGWTAFLSNMLFFTDFMGQ
Template.pdb      SGDRLS---SLRR--LGLVYFLYLFL-FSGLEYTLSFLTHQRFQ------
                  *.* :    *:.:   * *   * :* :* *    :**::  *

Model.pdb         IVYRGVEVGCWGLCINSVFSSLYSYFQKVLVSYIGLK-----G-------
Template.pdb      ------------------FSSLQQ---GKMFFLIGLTMATIQGAYARRIH
                                    **** .     :.  ***.     *

Model.pdb         -------------LYFTGYLLFGLGTGF--IGLFPNVYSTLVLCSLFGVM
Template.pdb      PGGEVAAVKRALLLLVPAFLLIGWGRSLPVLGLGL------LLYS---FA
                               * ...:**:* * .:  :**        :* *   .

Model.pdb         SSTLYTVPFNLITEYXRVRGKGMDCATLTCMVQLAQILVGGGLGFLVNTA
Template.pdb      AAVVVPCLSSVVAGYGSPGQKGTVMGTLRS------------LGALARAA
                  ::.: .   .::: *     **   .** .            ** *..:*

Model.pdb         GTVVVVVITASAVALIGCCFV----------------------
Template.pdb      GPLVAASVYW--LAGAQACFTTWSGLFLLPFFLLQKAENLYFQ
                  *.:*.. :    :*   .**.
```

**Fig 12:** Sequence alignment of SLC45A2 with percentage identity of 14 percent with its template, E-value=4.4e-26 and Score=153.21

## Variant mapping

UCSF Chimera[19] was used to generate the variant structures from the corresponding wild type structures. All the variants mapped into the wild type 3D structure are shown as in figure S13.

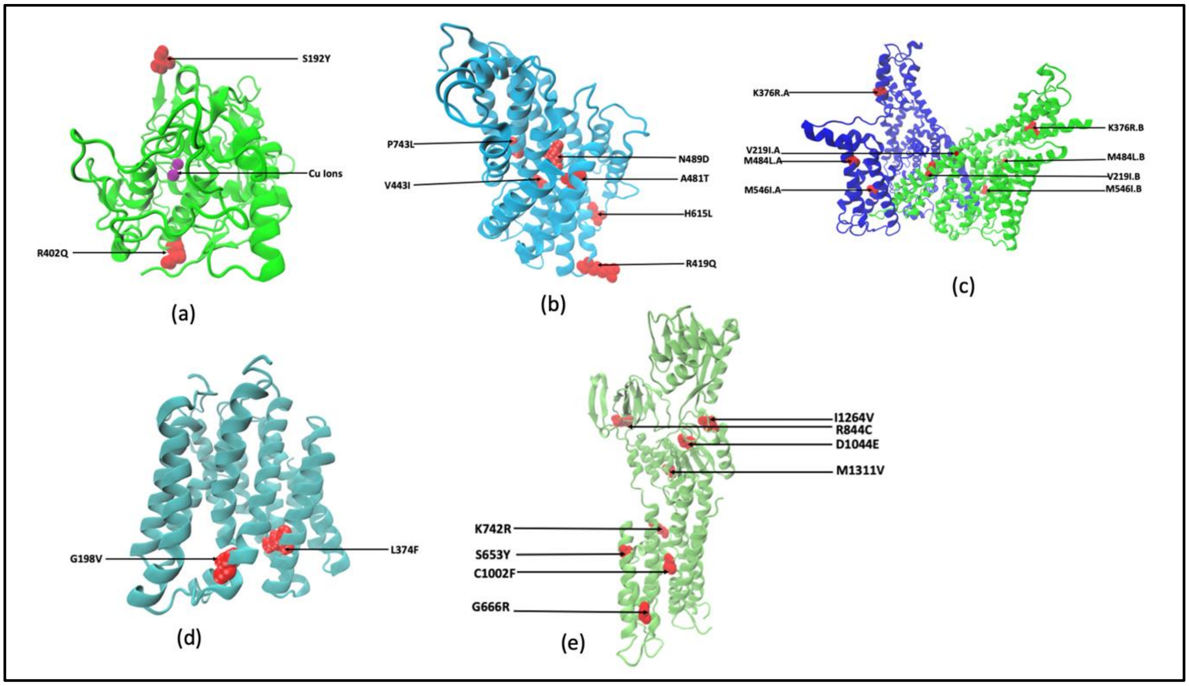


**Fig S13**. 3D structures with variants (shown in red color): (a) TYR; (b) OCA2; (c) TPC2; (d) SLC45A2; (e) ATP7A

## Generation of unfolded states

The unfolded structure of the wild type proteins were generated using "flexible meccano" approach[20, 21] (Fig. S14). The best model was selected from the ensemble of models having minimum solvent accessible surface area (SASA) measured using VMD[22]. The unfolded mutants were then generated using UCSF Chimera[19].

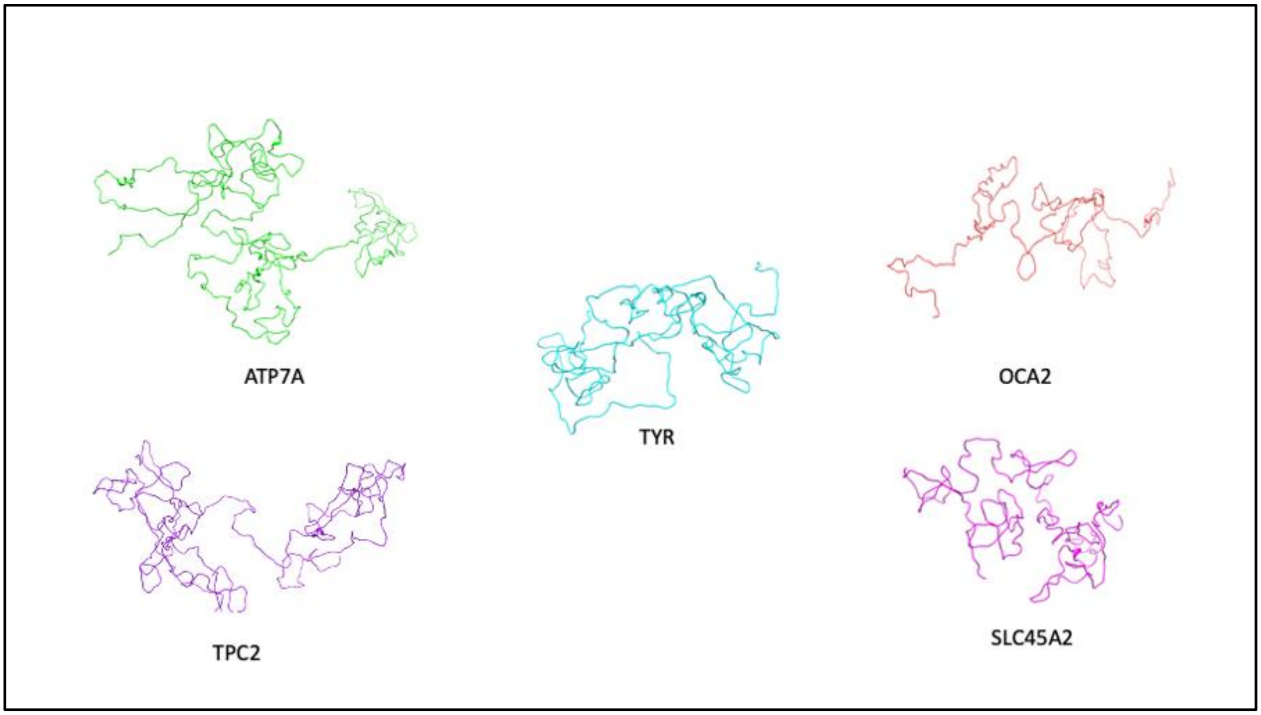


**Fig S14**. Unfolded structure of wild type proteins

**Table S1.** Calculated pKa's for the folded and unfolded structures.

| TYR | Folded/Unfolded | | |
|---|---|---|---|
| ResName | WT | S192Y | R402Q |
| ASP0042P | 3.38/3.71 | 3.72/3.71 | 3.49/3.71 |
| ASP0075P | 2.22/3.77 | 2.42/3.77 | 2.52/3.77 |
| ASP0076P | 2.45/3.99 | 3.19/3.99 | 3.1/3.97 |
| ASP0125P | 3.57/3.33 | 3.32/3.33 | 3.47/3.32 |
| ASP0132P | 3.58/3.78 | 3.71/3.78 | 3.58/3.77 |
| ASP0148P | 3.19/2.97 | 3.49/2.97 | 3.57/2.96 |
| ASP0169P | 3.76/3.88 | 3.47/3.88 | 3.15/3.88 |
| ASP0174P | 2.56/3.68 | 2.84/3.67 | 2.67/3.72 |
| ASP0186P | 2.95/3.49 | 3.43/3.49 | 3.09/3.53 |
| ASP0197P | 3.54/3.83 | 3.59/3.83 | 2.99/3.81 |
| ASP0199P | 3.51/3.54 | 2.32/3.54 | 1.92/3.54 |
| ASP0228P | 3.03/3.41 | 2.65/3.41 | 3.45/3.4 |
| ASP0237P | 3.36/3.83 | 2.43/3.83 | 2.36/3.85 |
| ASP0240P | 3.38/3.94 | 2.27/3.95 | 2.99/3.93 |
| ASP0245P | 2.84/3.35 | 3.42/3.35 | 3.58/3.32 |
| ASP0249P | 3.54/3.58 | 3.5/3.58 | 3.5/3.54 |
| ASP0305P | 3.56/3.54 | 3.49/3.54 | 3.28/3.52 |
| ASP0317P | 3.34/3.86 | 3.42/3.86 | 3.34/3.86 |
| ASP0333P | 2.59/3.05 | 3.41/3.06 | 2.82/3.04 |

| | | | |
|---|---|---|---|
| ASP0356P | 3.75/3.96 | 3.44/3.97 | 3.68/3.96 |
| ASP0383P | 2.03/3.76 | 2.25/3.76 | 2.27/3.76 |
| ASP0394P | 2.19/3.78 | 2.25/3.78 | 2.23/3.79 |
| ASP0437P | 3.16/3.41 | 2.52/3.41 | 2.53/3.41 |
| ASP0444P | 3.46/4.03 | 3.36/4.03 | 2.71/4.02 |
| ASP0448P | 2.83/3.76 | 3.34/3.76 | 3.56/3.75 |
| ASP0454P | undet/3.79 | 3.24/3.79 | 3.58/3.79 |
| GLU0032P | 3.05/3.81 | 3.99/3.81 | 3.59/3.8 |
| GLU0034P | 3.51/3.99 | 3.68/3.99 | 3.19/3.98 |
| GLU0078P | 3.23/4.37 | 2.75/4.37 | 2.86/4.35 |
| GLU0114P | 3.76/3.64 | 3.79/3.64 | 3.68/3.64 |
| GLU0130P | 3.38/3.95 | 3.02/3.95 | 2.63/3.95 |
| GLU0193P | 3.83/3.79 | 4.09/3.8 | 3.86/3.8 |
| GLU0203P | 3.14/3.83 | 3.28/3.83 | 3.23/3.84 |
| GLU0219P | 2.57/3.39 | 2.72/3.39 | 2.7/3.38 |
| GLU0221P | 3.1/4.01 | 2.83/4.01 | 3.02/4 |
| GLU0229P | 3.8/3.9 | 3.05/3.9 | 3.43/3.9 |
| GLU0242P | 3.51/3.77 | 3.54/3.78 | 3.49/3.76 |
| GLU0250P | 3.92/3.96 | 3.76/3.96 | 3.64/3.96 |
| GLU0280P | 3.74/3.45 | 3.61/3.45 | 3.54/3.42 |
| GLU0281P | 3.28/3.82 | 3.02/3.82 | 3.45/3.82 |
| GLU0294P | 2.97/3.05 | 3.44/3.05 | 2.94/3.05 |
| GLU0319P | 3.74/4 | 2.71/4 | 3.89/4 |

| | | | |
|---|---|---|---|
| GLU0328P | 2.02/3.59 | 2.89/3.58 | 2.03/3.56 |
| GLU0345P | 1.55/3.57 | 2.37/3.57 | 1.82/3.56 |
| GLU0398P | 2.09/3.85 | 2.08/3.85 | 2.53/3.98 |
| GLU0409P | 3.82/3.53 | 3.74/3.53 | 3.91/3.6 |
| GLU0413P | 3.73/3.77 | 3.59/3.77 | 3.85/3.77 |
| GLU0423P | 3.55/3.86 | 3.46/3.92 | 3.55/3.86 |
| HIS0019P | 6.33/6.5 | 6.38/6.5 | 6.37/6.5 |
| HIS0143P | 6.47/6.36 | 6.49/6.36 | 6.46/6.37 |
| HIS0180P | 5.38/6.32 | 5.9/6.32 | 7.04/6.35 |
| HIS0202P | 6.64/7.24 | 6.87/7.24 | 6.42/7.27 |
| HIS0211P | 5.9/6.38 | 5.95/6.38 | 5.71/6.38 |
| HIS0256P | 6.55/6.48 | 6.27/6.48 | 6.33/6.49 |
| HIS0285P | 6.68/6.67 | 6.83/6.67 | 6.66/6.67 |
| HIS0304P | 6.66/6.63 | 6.04/6.62 | 6.54/6.62 |
| HIS0363P | 6.04/6.5 | 6.39/6.5 | 6.35/6.51 |
| HIS0367P | 5.98/6.48 | 6.12/6.49 | 5.76/6.48 |
| HIS0389P | 5.64/6.52 | 6.08/6.52 | 5.87/6.52 |
| HIS0390P | 3.59/6.5 | 5.29/6.5 | 4.35/6.51 |
| HIS0404P | 6.56/6.3 | 6.68/6.3 | 6.58/6.46 |
| HIS0420P | 6.06/6.85 | 6.64/6.85 | 6.97/6.86 |

| TPC2 | Folded/Unfolded | | | | |
|---|---|---|---|---|---|
| ResName | WT | K376R | V219I | M546I | M484L |
| ASP0043A | 3.93/3.84 | 3.55/3.84 | 3.81/3.84 | 3.3/3.83 | 3.52/3.84 |
| ASP0047A | 3.37/3.86 | 3.05/3.86 | 3.27/3.86 | 2.71/3.83 | 3.53/3.86 |
| ASP0055A | 2.57/3.89 | 2.82/3.89 | 2.3/3.89 | 2.88/3.87 | 2.41/3.89 |
| ASP0067A | 3.04/3.75 | 2.95/3.75 | 2.84/3.75 | 3.04/3.75 | 3.25/3.75 |
| ASP0110A | 2.18/3.92 | 3.34/3.92 | 2.96/3.92 | 2.45/3.85 | 2.76/3.92 |
| ASP0139A | 2.99/3.06 | 2.71/3.06 | 3.26/3.06 | 2.88/2.69 | 3.15/3.06 |
| ASP0171A | 2.11/3.54 | 2.54/3.54 | 2.4/3.54 | 2.72/3.53 | 2.3/3.54 |
| ASP0244A | 3.54/3.63 | 3.53/3.63 | 3.92/3.63 | 3.48/3.58 | 2.83/3.63 |
| ASP0245A | 3.82/3.95 | 3.74/3.95 | 3.04/3.95 | 3.68/3.95 | 3.8/3.95 |
| ASP0248A | 3.76/3.46 | 3.53/3.46 | 3.62/3.46 | 3.77/3.12 | 3.67/3.46 |
| ASP0276A | 3.32/3.95 | 3.37/3.95 | 3.43/3.95 | 3.48/3.93 | 3.49/3.95 |
| ASP0372A | 3.14/3.24 | 2.99/3.22 | 3.23/3.24 | 2.99/3.23 | 3.14/3.24 |
| ASP0404A | 2.78/3.77 | 3.46/3.77 | 2.78/3.77 | 3.18/3.58 | 3.26/3.77 |
| ASP0435A | 3.23/3.89 | 3.49/3.89 | 3.03/3.89 | 3.11/3.87 | 2.71/3.89 |
| ASP0456A | 3.92/3.56 | 3.72/3.56 | 3.6/3.56 | 3.68/3.55 | 3.48/3.56 |
| ASP0458A | 3.83/3.31 | 3.76/3.31 | 3.63/3.31 | 2.79/3.06 | 3.12/3.31 |
| ASP0465A | 3.65/4 | 3.39/4 | 3.65/4 | 3.76/3.94 | 3.9/4 |
| ASP0466A | 3.03/4.09 | 3.49/4.09 | 3.51/4.09 | 3.53/3.63 | 3.49/4.09 |
| ASP0505A | 2.48/3.72 | 3.03/3.72 | 2.52/3.72 | 2.47/3.7 | 2.6/3.72 |
| ASP0542A | 3.28/3.79 | 3.38/3.79 | 2.43/3.79 | 3.16/3.77 | 2.05/3.79 |
| ASP0637A | 2.35/3.9 | 2.87/3.9 | 2.85/3.9 | 2.82/3.84 | 2.84/3.9 |

| | | | | | |
|---|---|---|---|---|---|
| ASP0638A | 1.95/3.97 | 2.35/3.97 | 2.77/3.97 | 2.2/3.97 | 3.33/3.97 |
| ASP0660A | 3.21/3.76 | 3.01/3.76 | 2.36/3.76 | 3.3/3.72 | 3.39/3.76 |
| GLU0054A | 2.17/3.85 | 2.97/3.85 | 2.44/3.85 | 3.42/3.82 | 2.32/3.85 |
| GLU0100A | 1.32/3.94 | 1.91/3.94 | 2.25/3.94 | 2.49/3.93 | 1.84/3.94 |
| GLU0119A | 3.85/3.54 | 4.02/3.54 | 3.95/3.54 | 3.92/3.5 | 3.97/3.54 |
| GLU0126A | 2.74/3.83 | 2.44/3.84 | 2.42/3.83 | 3.05/3.8 | 3.06/3.83 |
| GLU0129A | 1.75/3.9 | 1.79/3.9 | 2/3.9 | 1.98/3.87 | 1.93/3.9 |
| GLU0182A | 3.12/3.85 | 3.44/3.85 | 3.38/3.85 | 2.76/3.85 | 3.54/3.85 |
| GLU0215A | 3.84/3.97 | 3.8/3.97 | 3.75/3.97 | 3.65/3.93 | 3.82/3.97 |
| GLU0250A | 2.75/2.83 | 2.87/2.83 | 3.4/2.83 | 1.96/2.87 | 3.38/2.83 |
| GLU0260A | 3.7/4.1 | 3.59/4.1 | 3.74/4.1 | 2.95/4.07 | 3.72/4.1 |
| GLU0339A | 3.66/3.94 | 3.78/3.94 | 3.49/3.94 | 3.78/3.93 | 3.5/3.94 |
| GLU0347A | 3.75/3.91 | 4.04/3.91 | 3.87/3.91 | 3.86/3.91 | 3.83/3.91 |
| GLU0381A | 3.68/3.56 | 2.76/3.44 | 3.64/3.56 | 3.43/3.48 | 2.82/3.56 |
| GLU0394A | 3.55/3.85 | 3.44/3.85 | 3.74/3.85 | 2.68/3.83 | 3.72/3.85 |
| GLU0395A | 3.7/3.46 | 3.53/3.46 | 2.45/3.46 | 3.67/3.43 | 2.94/3.46 |
| GLU0402A | 3.44/4.01 | 2.14/4.01 | 2.83/4.01 | 2.66/3.97 | 3.01/4.01 |
| GLU0410A | 3.18/3.93 | 3.83/3.93 | 2.36/3.93 | 3.41/3.88 | 3.76/3.93 |
| GLU0416A | 3.8/3.53 | 3.78/3.53 | 3.35/3.53 | 3.55/3.53 | 3.37/3.53 |
| GLU0463A | 3.87/3.62 | 3.89/3.62 | 3.68/3.62 | 3.74/3.44 | 3.8/3.62 |
| GLU0483A | 2.02/3.96 | 2.28/3.96 | 2.53/3.96 | 3.1/3.94 | 3.5/3.96 |
| GLU0516A | 3.51/3.97 | 3.95/3.97 | 3.95/3.97 | 3.93/3.97 | 3.85/3.97 |
| GLU0533A | 3.58/3.45 | 3.5/3.45 | 2.91/3.45 | 3.78/3.2 | 3.49/3.45 |

| | | | | | |
|---|---|---|---|---|---|
| GLU0627A | 2.8/3.97 | 3.44/3.97 | 3.49/3.97 | 3.02/3.96 | 3.78/3.97 |
| GLU0630A | 3.41/3.99 | 4.01/3.99 | 3.77/3.99 | 4.11/3.97 | 4.01/3.99 |
| GLU0695A | 2.55/4 | 2.66/4 | 2.82/4 | 2.65/4.01 | 2.47/4 |
| HIS0064A | 6.5/6.46 | 6.05/6.46 | 6.3/6.46 | 6.15/6.47 | 6.28/6.46 |
| HIS0151A | 6/6.48 | 5.81/6.47 | 5.98/6.48 | 5.92/6.47 | 5.69/6.48 |
| HIS0181A | 6.82/6.5 | 6.68/6.5 | 6.71/6.5 | 6.74/6.49 | 6.73/6.5 |
| HIS0226A | 6.22/6.73 | 6.22/6.73 | 6.02/6.73 | 6.17/6.73 | 6.28/6.73 |
| HIS0375A | 6.18/6.55 | 6.52/6.53 | 6.35/6.55 | 6.51/6.53 | 6.46/6.55 |
| HIS0411A | 6.04/6.49 | 5.96/6.49 | 5.96/6.49 | 6.26/6.49 | 6.64/6.49 |
| HIS0431A | 6.61/6.25 | 6.5/6.25 | 6/6.25 | 6.46/6.24 | 6.21/6.25 |
| HIS0527A | 6.65/6.49 | 5.82/6.49 | 6.66/6.49 | 6.62/6.49 | 6.47/6.49 |
| HIS0699A | 5.99/6.88 | 6.26/6.88 | 6.33/6.88 | 5.86/6.86 | 5.76/6.88 |

| SLC45A2 | Folded/Unfolded | | |
|---|---|---|---|
| ResName | WT | G198V | I374F |
| ASP0093X | 1.73/3.31 | 2.19/3.31 | 2.19/3.31 |
| ASP0153X | 4.16/3.82 | 4.2/3.82 | 4.2/3.82 |
| ASP0157X | 3.92/3.94 | 4.07/3.94 | 4.07/3.94 |
| ASP0160X | 2.05/3.78 | 2.62/3.78 | 2.62/3.78 |
| ASP0169X | 3.08/3.86 | 3.49/3.86 | 3.49/3.86 |
| ASP0175X | 2.87/3.19 | 3.25/3.19 | 3.25/3.19 |

| | | | |
|---|---|---|---|
| ASP0201X | 3.99/3.99 | 4.02/3.99 | 4.02/3.99 |
| ASP0257X | 3.79/3.98 | 3.83/3.98 | 3.83/3.98 |
| ASP0263X | 3.66/3.84 | 3.84/3.84 | 3.84/3.84 |
| ASP0340X | 3.69/3.7 | 3.7/3.7 | 3.7/3.7 |
| ASP0475X | 2.64/3.72 | 3.49/3.72 | 3.49/3.72 |
| GLU0026X | 3.85/3.81 | 3.97/3.81 | 3.97/3.81 |
| GLU0046X | 3.43/3.83 | 3.6/3.83 | 3.6/3.83 |
| GLU0052X | 4.41/3.74 | 4.46/3.74 | 4.46/3.74 |
| GLU0177X | 3.04/3.45 | 3.72/3.45 | 3.72/3.45 |
| GLU0206X | 3.65/2.88 | 4.04/2.88 | 4.04/2.88 |
| GLU0214X | 4.04/3.84 | 4.19/3.84 | 4.19/3.84 |
| GLU0239X | 3.73/3.77 | 3.78/3.77 | 3.78/3.77 |
| GLU0244X | 3.62/3.82 | 3.79/3.82 | 3.79/3.82 |
| GLU0267X | 3.72/3.99 | 4.01/3.99 | 4.01/3.99 |
| GLU0272X | 2.96/3.66 | 3.07/3.68 | 3.07/3.68 |
| GLU0368X | 3.96/3.82 | 3.98/3.82 | 3.98/3.82 |
| GLU0448X | 3.2/3.64 | 3.68/3.64 | 3.68/3.64 |
| HIS0038X | 5.55/6.48 | 6.24/6.48 | 5.55/6.48 |
| HIS0094X | 6.24/6.31 | 6.54/6.31 | 6.03/6.31 |
| HIS0173X | 6.24/6.13 | 6.35/6.13 | 6.17/6.13 |
| HIS0181X | 6.03/6.26 | 6.36/6.26 | 6.3/6.26 |
| HIS0183X | 6.22/5.69 | 6.69/5.69 | 6.03/5.69 |
| HIS0204X | 6.76/6.72 | 6.69/6.72 | 6.97/6.72 |

| | | | |
|---|---|---|---|
| HIS0233X | 4.84/6.46 | 5.63/6.46 | 6.07/6.46 |
| HIS0316X | 6.22/6.33 | 6.73/6.33 | 6.25/6.33 |
| HIS0324X | 6.32/6.68 | 6.51/6.68 | 6.12/6.68 |
| HIS0450X | 6.6/6.34 | 5.66 /6.34 | 6.61/6.34 |

| OCA2 | Folded/Unfolded | | | | | | |
|---|---|---|---|---|---|---|---|
| ResName | WT | V433I | R419Q | P743L | N489D | H615L | A481T |
| ASP0372U | 3.66/3.85 | 3.63/3.56 | 3.05/3.94 | 3.64/3.83 | 3.48/3.94 | 3.02/3.86 | 3.73/3.88 |
| ASP0384U | 4.06/4.46 | 3.92/4.01 | 4.14/3.94 | 4.04/3.99 | 3.98/3.94 | 3.91/4.02 | 3.98/4.08 |
| ASP0408U | 3.54/4.03 | 3.19/3.53 | 3.56/3.93 | 3.44/3.79 | 3.36/3.95 | 3.74/3.84 | 3.56/3.77 |
| ASP0441U | 3.05/4.16 | 2.74/2.91 | 1.97/3.94 | 2.68/3.88 | 3.37/3.94 | 3.47/3.79 | 2.94/3.83 |
| ASP0463U | 2.51/3.85 | 2.96/2.81 | 2.35/3.93 | 2.98/3.97 | 2.69/3.94 | 2.51/4.16 | 2.73/3.71 |
| ASP0486U | 3.43/4.01 | 3.51/3.73 | 3.47/3.95 | 3.38/3.98 | 2.52/3.94 | 3.59/3.87 | 3/3.99 |
| ASP0504U | 3.48/4.13 | 3.46/3.29 | 3.26/4.04 | 3.49/3.76 | 3.46/3.94 | 3.69/3.68 | 3.52/3.75 |
| ASP0601U | 2.83/4.09 | 3.42/2.77 | 3.11/3.79 | 2.54/3.53 | 2.7/3.96 | 3.25/3.76 | 2.46/3.32 |
| ASP0619U | 2.99/3.73 | 2.2/2.81 | 2.86/3.89 | 2.98/3.89 | 3.22/3.94 | 2.87/3.64 | 2.35/3.83 |
| ASP0649U | 3.91/3.79 | 3.5/3.85 | 3.56/3.76 | 3.95/3.75 | 3.8/3.93 | 3.18/3.71 | 3.7/3.63 |
| ASP0666U | 4.03/4 | 3.79/4.1 | 3.98/3.93 | 3.97/4.3 | 4.04/3.94 | 4.07/3.46 | 4.02/3.44 |
| ASP0669U | 3.57/3.64 | 3.42/3.71 | 3.63/3.94 | 3.75/3.99 | 3.5/3.94 | 3.8/3.87 | 3.54/4.04 |
| ASP0740U | 3.49/3.99 | 3.05/4.05 | 3.6/4.06 | 2.33/3.87 | 3.4/3.93 | 2.53/3.97 | 3.5/3.87 |
| ASP0758U | 3.93/4.05 | 3.5/3.99 | 2.98/4.02 | 3.9/3.67 | 4.11/3.96 | 3.53/3.77 | 3.88/3.85 |

| | | | | | | | |
|---|---|---|---|---|---|---|---|
| GLU0328U | 3.77/4.19 | 3.9/3.54 | 3.06/4.01 | 3.5/3.87 | 3.57/3.92 | 3.57/4.01 | 3.48/4.03 |
| GLU0348U | 3.32/3.8 | 3.7/3.2 | 3.53/4.01 | 3.2/3.79 | 3.3/4.02 | 3.87/3.52 | 3.01/3.97 |
| GLU0381U | 3.81/4.03 | 3.98/3.48 | 3.59/4.01 | 3.91/4.02 | 3.8/4.04 | 3.9/3.93 | 3.89/4.08 |
| GLU0386U | 3.31/4.68 | 3.63/3.86 | 4.25/4.01 | 3.55/4.07 | 3.43/4 | 3.84/3.91 | 3.69/4.05 |
| GLU0403U | 3.95/4.26 | 3.54/3.76 | 3.78/4 | 3.62/4.17 | 3.92/4.02 | 3.65/3.85 | 3.6/3.96 |
| GLU0458U | 3.35/4.09 | 3.73/3.96 | 3.4/4 | 3.85/3.87 | 3.61/4.03 | 3.94/4.04 | 3.75/3.79 |
| GLU0471U | 2.5/4.21 | 3.99/4.01 | 4.03/4.02 | 3.92/3.96 | 3.87/4 | 4.13/3.98 | 2.67/4.19 |
| GLU0497U | 3.7/3.96 | 3.6/3.23 | 3.61/4 | 3.24/3.97 | 3.81/4 | 3.42/3.74 | 3.59/3.95 |
| GLU0540U | 3.76/4.4 | 3.72/3.51 | 3.68/4.03 | 3.91/4 | 3.44/4.02 | 3.89/4.05 | 3.43/4.01 |
| GLU0543U | 3.55/4.38 | 3.75/3.82 | 2.31/4.02 | 3.75/3.85 | 3.98/4 | 3.78/3.84 | 3.76/3.26 |
| GLU0546U | 2.78/3.92 | 3.26/3.2 | 3.55/4 | 2.83/3.94 | 3.08/4 | 3.28/3.98 | 2.94/3.96 |
| GLU0550U | 3.16/3.63 | 2.92/3.8 | 3.77/4.03 | 3.52/3.65 | 2.65/4 | 3.62/3.78 | 3.56/3.71 |
| GLU0567U | 2.27/4.05 | 3.27/3.58 | 3.02/4.01 | 3.09/3.95 | 3.74/4 | 3.86/3.66 | 3.93/3.72 |
| GLU0568U | 2.25/4 | 4.03/3.6 | 3.41/4.03 | 3.34/3.77 | 2.34/4 | 1.89/3.43 | 3.94/3.49 |
| GLU0583U | 3.83/4.12 | 3.73/3.7 | 3.08/4.07 | 3.75/3.78 | 3.39/4.02 | 3.47/3.54 | 3.63/3.56 |
| GLU0600U | 3.82/4.05 | 2.8/3.98 | 2.71/4.01 | 3.78/4.06 | 3.07/4 | 3.79/3.42 | 3.73/3.55 |
| GLU0605U | 2.5/4.01 | 2.46/3.22 | 3.77/3.95 | 2.33/3.68 | 2.46/4.04 | 2.85/3.2 | 2.82/3.72 |
| GLU0610U | 3.19/3.98 | 3.87/3.56 | 3.82/3.97 | 2.4/3.97 | 2.01/4 | 2.87/3.8 | 2.43/3.84 |
| GLU0671U | 3.98/3.41 | 3.96/3.89 | 3.79/3.86 | 3.88/3.04 | 3.98/4.02 | 3.91/3.96 | 3.98/4.05 |
| GLU0678U | 3.22/4.15 | 2.49/3.31 | 3.62/3.97 | 3.41/3.85 | 3.72/4 | 2.99/3.82 | 3.55/3.94 |
| GLU0693U | 3.07/4 | 3.42/2.81 | 2.54/3.63 | 3.07/3.82 | 2.54/3.84 | 3/3.62 | 2.81/4.04 |
| GLU0702U | 3.96/4.21 | 4.03/3.84 | 3.32/3.99 | 3.6/4.03 | 4.15/3.68 | 3.88/4.01 | 3.78/3.93 |
| GLU0706U | 3.22/4.29 | 3.89/4.08 | 4.15/3.77 | 3.97/3.83 | 4.12/3.96 | 4.05/4.12 | 4.08/3.78 |

| | | | | | | | |
|---|---|---|---|---|---|---|---|
| GLU0717U | 4.05/3.56 | 3.91/4.05 | 4.08/3.92 | 4.06/3.83 | 4.15/4.04 | 4.01/3.95 | 4.01/3.82 |
| GLU0718U | 3.45/3.5 | 3.97/4.07 | 4.04/4.33 | 4.03/3.93 | 4.24/3.97 | 4.02/4.15 | 4.01/3.53 |
| GLU0760U | 3.29/3.8 | 3.98/4.08 | 3.92/3.98 | 3.92/4.01 | 3.94/3.8 | 4/3.99 | 3.8/4 |
| GLU0798U | 4.07/4.56 | 3.65/3.51 | 3.96/3.96 | 3.81/3.82 | 3.7/3.74 | 3.02/3.97 | 3.78/3.89 |
| GLU0808U | 1.92/3.53 | 1.98/1.74 | 2.46/6.48 | 1.97/3.65 | 1.79/3.92 | 2.35/3.83 | 2.3/3.95 |
| HIS0351 | 6.4/6.64 | 6.2/5.97 | 6.1/6.48 | 6.1/6.62 | 6.3/3.76 | 6.2/6.82 | 6.5/6.5 |
| HIS0378 | 6/6.85 | 6.9/6.62 | 6/6.49 | 6.7/6.51 | 6.5/6.49 | 6.1/6.33 | 6.8/6.37 |
| HIS0511 | 6.8/6.53 | 6.3/6.66 | 6.4/6.49 | 6.5/6.55 | 6.6/6.49 | 6.9/6.39 | 6.6/6.26 |
| HIS0549 | 6.5/6.33 | 6.2/6.54 | 6.3/6.49 | 6.3/6.44 | 6.2/6.48 | 6.4/6.68 | 6.2/6.29 |
| HIS0552 | 6.2/7.12 | 6.3/6.44 | 6.3/6.5 | 5.8/6.81 | 6.5/6.49 | 6.3/6.37 | 6.4/6.8 |
| HIS0584 | 6.1/6.38 | 6/5.6 | 6.3/6.49 | 6.3/6.37 | 6.1/6.49 | 6.5/6.67 | 5.7/6.52 |
| HIS0591 | 6.2/6.58 | 6.2/5.8 | 6.2/6.49 | 5.7/5.98 | 6.1/6.49 | 6.1/5.95 | 6.1/6.15 |
| HIS0594 | 5.9/5.82 | 6.2/6.01 | 6.1/6.27 | 6.3/6.51 | 6.2/6.49 | 6.1/5.83 | 6.6/6.58 |
| HIS0647 | 6.2/6.76 | 6.6/7.02 | 6.6/7.01 | 6.3/6.54 | 6.7/6.5 | 6.8/6.52 | 6.2/6.17 |
| HIS0668 | 6.8/6.9 | 6.9/6.76 | 7.1/6.72 | 6.6/6.72 | 6.5/6.49 | 6.8/6.38 | 6.4/6.54 |
| HIS0675 | 6.6/6.64 | 6.7/6.72 | 6.8/6.86 | 6.7/6.29 | 6.7/6.49 | 6.7/6.5 | 6.7/6.52 |
| HIS0697 | 6.6/6.59 | 6.6/6.76 | 6.7/6.55 | 6.6/6.21 | 6.8/6.18 | 6.7/6.64 | 6.7/6.26 |
| HIS0699 | 6.5/6.55 | 6.1/7.03 | 6.6/6.78 | 6.9/6.4 | 7.1/6.9 | 6.5/6.84 | 6.4/6.29 |
| HIS0757 | 6.7/7.22 | 7.1/6.59 | 6.7/6.67 | 6.8/7 | 7/6.51 | 6.7/6.53 | 6.9/6.54 |
| HIS0800 | 6.5/6.77 | 6.4/6.46 | 6.7/10.77 | 6.6/6.63 | 6.6/6.76 | 6.5/10.66 | 6.4/6.82 |

| ATP7A | Folded/Unfolded | | | | | | | |
|---|---|---|---|---|---|---|---|---|
| ResName | WT | C1002F | D1044E | G666R | I1264V | K742R | M1311V | R844C | S653Y |
| ASP0675P | 3.52/3.47 | 3.23/3.53 | 3.19/3.35 | 3.52/3.47 | 3.73/3.35 | 3.36/3.2 | 3.65/3.35 | undet/3.55 | 3.54/3.47 |
| ASP0747P | 2.46/2.23 | 2.75/2.7 | 2.99/2.68 | 2.91/3.1 | 3.07/3.78 | 3.18/2.81 | 3.15/3.78 | 2.52/2.28 | 2.58/2.7 |
| ASP0782P | 3.37/3.65 | 3.53/3.35 | 3.62/3.69 | 3.46/3.48 | 3.32/3.91 | 3.51/3.49 | 3.7/3.91 | 3.55/3.62 | 3.75/3.77 |
| ASP0826P | 3.38/3.31 | 3.48/3.13 | 3.58/3.89 | 3.43/3.84 | 3.79/3.93 | 3.52/3.9 | 3.89/3.93 | 3.01/3.05 | 4/3.23 |
| ASP0828P | 3.95/4 | 3.94/3.99 | 3.98/3.96 | 4.1/3.96 | 3.86/3.98 | 3.93/3.96 | 3.57/3.98 | 3.79/3.73 | 3.99/4 |
| ASP0838P | 3.95/3.98 | 3.91/3.9 | 3.95/3.84 | 3.87/3.95 | 3.95/4.11 | 3.82/3.94 | 3.93/4.11 | 3.93/3.91 | 3.97/3.88 |
| ASP0846P | 3.58/3.76 | 3.72/3.67 | 3.81/3.71 | 3.59/3.77 | 3.68/2.98 | 3.7/3.71 | 3.74/2.98 | 3.84/3.85 | 3.77/3.69 |
| ASP0859P | 1.79/1.5 | 2.31/2.37 | 3.15/2.71 | 2.05/2.72 | 2.64/3.28 | 2.78/2.51 | 2.55/3.28 | 2.51/2.19 | 2.44/2.45 |
| ASP0870P | 3.74/3.54 | 3.48/3.49 | 3.66/3.68 | 3.93/3.53 | 3.5/3.95 | 3.41/3.59 | 3.57/3.95 | 2.73/2.83 | 3.89/3.91 |
| ASP0910P | 3.51/3.56 | 3.13/3.39 | 3.56/3.92 | 3.64/3.46 | 3.38/3.78 | 3.73/3.71 | 3.75/3.78 | 3.58/3.47 | 3.43/3.52 |
| ASP0935P | 3.35/3.44 | 2.66/2.93 | 2.53/2.32 | 3.11/3.08 | 3.28/3.77 | 3.35/3.08 | 3.08/3.77 | 2.23/2.19 | 2.65/2.57 |
| ASP1044P | 2.75/2.4 | 2.45/2.46 | 1.56/3.66 | 2.55/3.57 | 2.69/3.54 | 3.14/3.25 | 2.34/3.54 | 2.51/2.49 | 2.32/2.79 |
| ASP1101P | 3.74/3.84 | 3.73/3.58 | 3.57/3.67 | 3.44/3.53 | 3.64/3.74 | 3.76/3.76 | 3.67/3.74 | 3.85/3.84 | 3.7/3.7 |
| ASP1110P | 3.4/3.31 | 3.57/3.55 | 3.66/3 | 3.7/3.53 | 3.22/3.8 | 3.13/3.2 | 3.36/3.8 | 3.49/3.55 | 3.3/3.67 |
| ASP1139P | 3.34/3.84 | 3.7/3.69 | 3.75/3.65 | 3.67/3.48 | 3.58/3.86 | 3.78/3.85 | 3.39/3.86 | 3.73/3.7 | 3.77/3.57 |
| ASP1151P | 3.65/3.72 | 3.44/3.49 | 3.81/3.41 | 2.88/3.69 | 3.75/3.88 | 3.81/3.94 | 3.8/3.88 | 3.83/3.85 | 3.54/3.84 |
| ASP1166P | 3.81/3.82 | 3.81/3.85 | 3.49/3.59 | 3.48/3.78 | 3.56/4.26 | 3.71/3.46 | 3.75/4.26 | 3.82/3.76 | 3.93/3.84 |
| ASP1198P | 3.78/3.42 | 3.76/3.78 | 3.64/3.72 | 3.75/3.7 | 3.47/3.98 | 3.72/3.6 | 3.57/3.98 | 3.62/3.57 | 3.37/3.63 |
| ASP1201P | 3.74/3.71 | 3.76/3.76 | 3.6/2.16 | 3.65/3.15 | 3.62/3.82 | 3.7/3.72 | 3.69/3.82 | 3.77/3.69 | 3.7/3.81 |
| ASP1219P | 3.65/3.47 | 3.34/3.44 | 2.05/3.59 | 3.18/3.93 | 2.33/3.82 | 3.53/3.35 | 2.59/3.82 | 3.52/3.5 | 3.19/3.71 |
| ASP1220P | 3.04/2.7 | 3.35/3.62 | 3.58/2.69 | 3.97/2.96 | 3.95/3.77 | 2.97/3.41 | 2.99/3.77 | 3.33/3.35 | 3.13/3.4 |
| ASP1230P | 2.87/3.2 | 2.93/3.31 | 2.66/3.25 | 2.46/3.17 | 1.97/3.96 | 2.51/2.27 | 2.72/3.96 | 2.19/2.24 | 3.01/3.05 |
| ASP1256P | 2.72/2.62 | 2.97/2.94 | 3.61/3.62 | 2.98/2.68 | 2.53/3.67 | 3.05/2.81 | 2.72/3.67 | 2.82/2.92 | 3.01/2.99 |

| | | | | | | | | | |
|---|---|---|---|---|---|---|---|---|---|
| ASP1301P | 3.52/3.33 | 3.38/3.52 | 3.61/2.66 | 3.36/3.01 | 3.95/3.83 | 3.43/3.41 | 3.49/3.83 | 3.15/3.19 | 3.02/3.01 |
| ASP1305P | 2.42/2.65 | 2.89/2.65 | 2.74/3.31 | 2.55/3.56 | 2.3/3.56 | 2.46/2.23 | 2.74/3.55 | 2.46/1.94 | 2.11/1.98 |
| ASP1323P | 3.73/3.79 | 3.74/3.78 | 3.53/3.47 | 3.58/3.41 | 3.73/3.76 | 3.6/3.58 | 3.6/3.76 | 3.5/3.57 | 3.56/3.55 |
| ASP1330P | 2.71/2.8 | 2.67/2.38 | 3.56/3.16 | 3.46/3.68 | 3.44/3.8 | 3.29/3.24 | 2.65/3.8 | 2.88/2.79 | 3.41/3.49 |
| ASP1337P | 3.57/3.65 | 3.46/3.44 | 3.17/2.91 | 3.18/3.54 | 2.89/3.93 | 3.26/3.49 | 3.21/3.93 | 3.51/3.56 | 3.18/3.49 |
| ASP1340P | 2.71/2.98 | 2.31/2.43 | 2.94/2.71 | 3.67/2.7 | 3.05/3.7 | 3.67/3.84 | 2.66/3.7 | 2.9/3.09 | 3.47/3.41 |
| ASP1346P | 2.38/2.39 | 2.54/2.84 | 2.8/3.49 | 2.46/3.54 | 2.63/3.85 | 2.78/2.85 | 2.78/3.85 | 2.47/2.53 | 2.33/2.47 |
| GLU0646P | 3.37/2.39 | 3.83/3.66 | 3.9/3.87 | 3.56/3.84 | 3.78/4 | 3.13/2.72 | 3.72/4 | 3.72/3.73 | 3.21/3.56 |
| GLU0690P | 3.73/3.86 | 3.59/3.81 | 3.97/2.22 | 3.65/3.91 | 3.9/3.98 | 3.75/3.81 | 3.74/3.98 | 3.28/3.14 | 3.95/3.95 |
| GLU0691P | 3.83/3.96 | 3.75/4.01 | 2.18/3.44 | 3.75/2.72 | 3.75/3.99 | 2.78/3.69 | 3.94/3.99 | 3.88/3.95 | 3.77/3.94 |
| GLU0702P | 3.83/3.49 | 3.8/3.96 | 3.77/3.05 | 1.53/3.15 | 3.85/4.01 | 3.71/3.8 | 3.46/4.01 | 3.71/3.66 | 3.43/3.48 |
| GLU0771P | 3/3.46 | 3.42/3.48 | 3.26/3.03 | 3.62/2.69 | 2.86/3.65 | 3.12/2.76 | 3.42/3.65 | 3.45/3.64 | 3.23/3.05 |
| GLU0798P | 3.23/3.15 | 2.56/2.93 | 2.49/3.93 | 2.89/3.24 | 3.02/3.74 | 2.41/3.14 | 3.38/3.74 | 2.61/2.63 | 3.07/2.49 |
| GLU0807P | 3.71/3.83 | 4.01/3.93 | 4.14/4.01 | 3.84/4.02 | 3.24/3.81 | 4.05/3.92 | 3.95/3.81 | 2.84/3.13 | 3.69/3.29 |
| GLU0819P | 4.09/4.02 | 4.04/3.94 | 3.95/3.69 | 4.01/3.44 | 4.09/4.51 | 3.9/3.85 | 3.98/4.51 | 4.14/4.11 | 3.87/3.69 |
| GLU0834P | 3.47/3.52 | 3.26/3.45 | 3.52/4.13 | 3.12/4.04 | 2.97/3.94 | 3.06/3.48 | 3.27/3.94 | 3.49/3.47 | 3.29/3.21 |
| GLU0835P | 4.11/4.2 | 4.24/4.13 | 4.27/4.16 | 4.28/3.97 | 4.07/4.15 | 4.24/4.06 | 4.13/4.15 | 4.07/4.09 | 4.32/4.1 |
| GLU0840P | 4.06/4.05 | 4.02/4.04 | 4.01/3.74 | 4.02/3.74 | 3.94/4.01 | 3.96/4.08 | 4.01/4.01 | 4/3.98 | 3.98/3.98 |
| GLU0864P | 3.64/3.74 | 3.52/3.78 | 3.64/3.03 | 3.8/2.96 | 3.54/3.94 | 3.81/3.82 | 3.48/3.94 | 3.55/3.64 | 3.82/3.66 |
| GLU0871P | 3.33/2.81 | 3.17/3.23 | 2.92/4.06 | 2.83/3.76 | 3.3/3.81 | 3.34/3.43 | 3.45/3.81 | 3.67/3.86 | 3.49/3.46 |
| GLU0877P | 3.53/3.75 | 3.78/3.73 | 3.95/3.95 | 3.19/3.86 | 3.59/3.76 | 3.54/3.61 | 3.66/3.76 | 3.38/3.16 | 3.88/3.94 |
| GLU0921P | 3.76/3.76 | 3.8/3.91 | 3.57/3.55 | 3.32/4 | 3.74/3.73 | 3.97/4.02 | 3.45/3.73 | 3.72/3.87 | 3.75/3.13 |
| GLU0922P | 3.81/3.84 | 2.84/3.76 | 3.73/3.96 | 3.57/3.87 | 3.38/3.49 | 3.54/2.96 | 3.65/3.49 | 3.87/3.24 | 3.8/3.93 |
| GLU0965P | 3.26/4 | 4/3.78 | 4.15/3.96 | 3.87/4 | 2.75/3.97 | 4.01/3.73 | 1.99/3.97 | 4.01/3.88 | 2.69/2.77 |
| GLU0968P | 3.97/4.02 | 4.02/3.99 | 3.97/3.72 | 3.82/2.84 | 3.97/4.01 | 4/4.01 | 3.98/4.01 | 3.96/4.01 | 4.03/3.98 |

| | | | | | | | | | |
|---|---|---|---|---|---|---|---|---|---|
| GLU0982P | 3.76/3.14 | 3.54/3.51 | 3.7/3.14 | 3.51/3.54 | 2.7/3.77 | 2.88/2.77 | 3.02/3.77 | 3.52/3.54 | 3.69/3.82 |
| GLU1030P | 3.06/2.7 | 3.36/3.48 | 3.05/1.12 | 3.77/3.07 | 3.63/4.07 | 2.45/2.48 | 3.12/4.07 | 3.09/3.31 | 3.03/3.3 |
| GLU1033P | 3.1/2.99 | 3.05/3.08 | 2.23/2.1 | 3.03/2.39 | 2.94/3.83 | 3.12/3.1 | 3.23/3.83 | 2.76/2.78 | 2.83/2.93 |
| GLU1064P | 3.56/3.72 | 3.54/3.82 | 3.73/3.72 | 3.76/3.72 | 3.11/3.87 | 3.91/3.88 | 3.58/3.87 | 3.81/3.9 | 3.87/3.48 |
| GLU1081P | 2.27/3.29 | 2.85/3.18 | 3.5/3.49 | 3.33/3.34 | 2.63/4.01 | 3.39/3.53 | 3.47/4.01 | 3.47/3.46 | 2.36/2.3 |
| GLU1085P | 2.99/2.85 | 3.85/3.94 | 3.91/3.96 | 3.83/3.98 | 3.98/3.68 | 3.68/3.9 | 3.97/3.68 | 3.79/4.01 | 3.84/3.85 |
| GLU1099P | 3.85/3.79 | 3.56/3.67 | 3.85/4 | 3.97/3.97 | 3.97/3.23 | 3.96/3.97 | 3.93/3.23 | 3.56/3.58 | 3.87/3.98 |
| GLU1103P | 3.84/3.73 | 3.3/3.86 | 3.3/2.64 | 3.57/3.16 | 2.97/3.92 | 3.13/3.86 | 3.27/3.92 | 3.81/3.75 | 3.54/2.65 |
| GLU1127P | 3.16/3.73 | 3.86/3.6 | 3.84/3.87 | 3.99/3.83 | 3.84/3.87 | 3.73/3.67 | 3.83/3.87 | 3.79/3.72 | 2.75/3.64 |
| GLU1138P | 3.77/3.93 | 3.74/3.71 | 3.78/3.97 | 4/3.94 | 3.74/3.95 | 3.99/4.04 | 3.98/3.95 | 3.82/3.78 | 3.82/3.94 |
| GLU1155P | 3.74/3.85 | 4.01/4 | 3.96/3.85 | 3.75/3.66 | 4.07/4.01 | 3.96/3.68 | 3.02/4.01 | 3.69/3.85 | 4.04/3.97 |
| GLU1186P | 3.4/2.45 | 2.25/3.19 | 2.98/2.8 | 2.36/2.85 | 2.48/3.84 | 3.41/3.15 | 3.46/3.84 | 3.54/3.43 | 3.49/3.47 |
| GLU1205P | 3.52/3.42 | 3.27/3.22 | 2.86/2.93 | 3.23/3.03 | 2.98/3.22 | 2.98/3.18 | 3.25/3.22 | 3.05/2.79 | 3.47/3.06 |
| GLU1207P | 2.73/2.68 | 3.07/3.38 | 2.93/3.49 | 2.5/2.46 | 3.34/3.47 | 3.12/3.46 | 3.22/3.47 | 2.74/3.33 | 3.39/3.41 |
| GLU1221P | 2.91/3.67 | 4.12/4.1 | 2.94/3.54 | 4.07/4.07 | 3.15/3.55 | 4.06/3.71 | 4.09/3.55 | 4.11/4.12 | 3.53/3.68 |
| GLU1235P | 3.92/3.82 | 3.49/3.38 | 2.58/2.48 | 3.52/3.4 | 3.81/4.04 | 3.59/3.56 | 3.83/4.04 | 3.53/3.61 | 3.54/3.74 |
| GLU1237P | 3.92/3.92 | 3.59/3.76 | 3.8/3.93 | 3.77/3.83 | 3.78/4 | 3.85/3.67 | 3.56/4 | 3.75/3.75 | 3.78/3.84 |
| GLU1249P | 2.35/3.11 | 2.89/2.74 | 2.98/2.62 | 3.04/2.99 | 3.15/3.32 | 3.15/2.85 | 3.66/3.32 | 2.52/2.8 | 2.86/2.72 |
| GLU1276P | 3.42/3.52 | 3.51/3.58 | 3.61/3.6 | 3.49/3.57 | 3.46/3.96 | 3.6/3.67 | 3.36/3.96 | 3.34/3.08 | 3.48/3.55 |
| GLU1291P | 3.73/3.79 | 3.88/3.92 | 3.51/3.49 | 4.02/3.21 | 3.34/3.95 | 3.68/3.78 | 3.89/3.95 | 3.3/3.55 | 3.42/3.02 |
| GLU1292P | 3.55/3.81 | 3.93/3.8 | 3.94/3.63 | 3.97/3.82 | 3.83/3.77 | 3.77/3.6 | 3.82/3.77 | 3.55/3.94 | 3.56/3.72 |
| GLU1327P | 3.53/2.95 | 3.72/3.63 | 3.52/3.77 | 3.18/3.51 | 3.7/3.7 | 3.09/2.92 | 3.36/3.7 | 4/4 | 3.06/2.55 |
| HIS0676P | 6.05/6.51 | 6.65/6.41 | 6.33/6.49 | 6.66/6.57 | 6.71/6.51 | 6.66/6.69 | 6.46/6.51 | 6.46/6.52 | 6.29/6.48 |
| HIS0677P | 6.48/6.42 | 6.46/6.34 | 5.95/4.17 | 6.32/6.52 | 6.53/6.95 | 6.04/6.33 | 6.19/6.95 | 6.2/6.29 | 6.19/6.33 |
| HIS0682P | 5.54/5.97 | 6.46/6.56 | 6.46/6.33 | 6.42/6.5 | 6.41/6.85 | 6.45/6.34 | 6.45/6.85 | 6.48/6.43 | 6.46/6.48 |

| | | | | | | | | | |
|---|---|---|---|---|---|---|---|---|---|
| HIS0683P | 6.49/6.46 | 6.51/6.28 | 6.04/6.17 | 6.34/6.54 | 6.04/6.54 | 6.49/6.45 | 6.2/6.54 | 6.34/6.33 | 6.25/6.28 |
| HIS0696P | 6.64/6.51 | 6.45/6.52 | 6.57/6.55 | 6.67/6.51 | 6.66/6.47 | 6.66/6.5 | 6.71/6.47 | 6.5/6.49 | 6.53/6.54 |
| HIS0741P | 6.37/6.46 | 6.32/6.46 | 6.14/6.18 | 6.51/6.48 | 6.17/6.49 | 6.14/6.18 | 6.3/6.49 | 6.17/6.13 | 6.21/5.62 |
| HIS0799P | 6.3/6.38 | 6.29/6.35 | 6.35/6.26 | 5.99/6.54 | 6.47/6.33 | 6.32/6.16 | 6.38/6.33 | 6.3/6.24 | 6.34/6.52 |
| HIS0866P | 6.46/6.45 | 5.85/5.81 | 5.53/6.25 | 5.63/6.16 | 5.99/6.47 | 6.28/6.36 | 6.18/6.47 | 6.29/6.31 | 6.29/6.34 |
| HIS0906P | 6.47/6.51 | 6.54/6.25 | 6.32/6.66 | 6.49/6.3 | 6.78/6.06 | 6.62/6.64 | 6.36/6.06 | 6.56/6.53 | 6.47/6.67 |
| HIS1036P | 5.67/5.81 | 5.76/5.78 | 5.5/5.78 | 5.77/5.7 | 5.99/6.79 | 5.96/6.11 | 5.88/6.79 | 5.71/5.77 | 5.75/5.37 |
| HIS1051P | 6.37/6.46 | 6.44/6.29 | 6.3/6.22 | 6.34/6.34 | 6.47/6.24 | 6.48/6.44 | 6.33/6.24 | 6.27/6.2 | 6.05/6.34 |
| HIS1070P | 6.17/5.93 | 5.94/6.29 | 5.76/6.06 | 6.14/6.17 | 5.98/6.44 | 6.04/6.06 | 6.18/6.44 | 6.07/6.14 | 5.48/5.57 |
| HIS1071P | 6.31/6.68 | 6.52/6.56 | 6.6/6.53 | 6.36/6.18 | 6.5/6.48 | 6.16/6.22 | 6.51/6.48 | 6.22/6.47 | 6.46/6.35 |
| HIS1086P | 6.53/6.34 | 6.47/6.65 | 6.64/6.56 | 6.2/6.33 | 6.01/6.27 | 6.57/6.27 | 6.15/6.27 | 6.38/6.45 | 6.55/6.65 |
| HIS1131P | 6.49/6.48 | 5.95/6.45 | 6.56/6.54 | 6.65/6.68 | 6.61/6.46 | 6.74/6.53 | 6.52/6.46 | 6.35/6.26 | 6.48/6.24 |
| HIS1206P | 6.25/6.15 | 6.06/5.96 | 6.06/6.01 | 6.13/5.93 | 5.93/6.71 | 5.99/6.06 | 6.18/6.71 | 6.18/6.17 | 6.16/6.02 |
| HIS1241P | 6.49/6.51 | 6.5/6.54 | 6.32/6.46 | 6.49/6.45 | 6.46/6.51 | 6.49/6.46 | 6.54/6.51 | 6.34/6.35 | 6.28/6.46 |
| HIS1281P | 6.12/5.52 | 5.83/6.11 | 6.22/6.26 | 5.76/6.45 | 6.05/6.47 | 6.33/6.29 | 6.05/6.47 | 5.36/5.98 | 6.2/6.27 |

* undet = Indetermined

| pKa (Folded-Unfolded) | Color code |
|---|---|
| $0.5 \leq \Delta pKa < 1.0$ | |
| $\Delta pKa \geq 1.0$ | |
| $-1.0 < \Delta pKa \leq -0.5$ | |
| $\Delta pKa \leq -1.0$ | |

Reference:


1. Cao, H.; Wang, J.; He, L.; Qi, Y.; Zhang, J. Z., DeepDDG: predicting the stability change of protein point mutations using neural networks. *Journal of chemical information and modeling* **2019,** *59* (4), 1508-1514.
2. Capriotti, E.; Fariselli, P.; Casadio, R., I-Mutant2. 0: predicting stability changes upon mutation from the protein sequence or structure. *Nucleic acids research* **2005,** *33* (suppl_2), W306-W310.
3. Chen, C.-W.; Lin, J.; Chu, Y.-W. In *iStable: off-the-shelf predictor integration for predicting protein stability changes*, BMC bioinformatics, Springer: 2013; p S5.
4. Cheng, J.; Randall, A.; Baldi, P., Prediction of protein stability changes for single-site mutations using support vector machines. *Proteins: Structure, Function, and Bioinformatics* **2006,** *62* (4), 1125-1132.
5. Getov, I.; Petukh, M.; Alexov, E., SAAFEC: predicting the effect of single point mutations on protein folding free energy using a knowledge-modified MM/PBSA approach. *International journal of molecular sciences* **2016,** *17* (4), 512.
6. Pandurangan, A. P.; Ochoa-Montaño, B.; Ascher, D. B.; Blundell, T. L., SDM: a server for predicting effects of mutations on protein stability. *Nucleic acids research* **2017,** *45* (W1), W229-W235.
7. Pires, D. E.; Ascher, D. B.; Blundell, T. L., mCSM: predicting the effects of mutations in proteins using graph-based signatures. *Bioinformatics* **2014,** *30* (3), 335-342.
8. Pires, D. E.; Ascher, D. B.; Blundell, T. L., DUET: a server for predicting effects of mutations on protein stability using an integrated computational approach. *Nucleic acids research* **2014,** *42* (W1), W314-W319.
9. Savojardo, C.; Fariselli, P.; Martelli, P. L.; Casadio, R., INPS-MD: a web server to predict stability of protein variants from sequence and structure. *Bioinformatics* **2016,** *32* (16), 2542-2544.
10. Guex, N.; Peitsch, M. C., SWISS-MODEL and the Swiss-Pdb Viewer: an environment for comparative protein modeling. *electrophoresis* **1997,** *18* (15), 2714-2723.
11. Consortium, U., UniProt: a hub for protein information. *Nucleic acids research* **2015,** *43* (D1), D204-D212.
12. Lai, X.; Wichers, H. J.; Soler-Lopez, M.; Dijkstra, B. W., Structure of human tyrosinase related protein 1 reveals a binuclear zinc active site important for melanogenesis. *Angewandte Chemie International Edition* **2017,** *56* (33), 9812-9815.
13. Kelley, L. A.; Mezulis, S.; Yates, C. M.; Wass, M. N.; Sternberg, M. J., The Phyre2 web portal for protein modeling, prediction and analysis. *Nature protocols* **2015,** *10* (6), 845-858.
14. Mancusso, R.; Gregorio, G. G.; Liu, Q.; Wang, D.-N., Structure and mechanism of a bacterial sodium-dependent dicarboxylate transporter. *Nature* **2012,** *491* (7425), 622-626.
15. She, J.; Zeng, W.; Guo, J.; Chen, Q.; Bai, X.-c.; Jiang, Y., Structural mechanisms of phospholipid activation of the human TPC2 channel. *Elife* **2019,** *8*, e45222.
16. Nomura, N.; Verdon, G.; Kang, H. J.; Shimamura, T.; Nomura, Y.; Sonoda, Y.; Hussien, S. A.; Qureshi, A. A.; Coincon, M.; Sato, Y., Structure and mechanism of the mammalian fructose transporter GLUT5. *Nature* **2015,** *526* (7573), 397-401.

17. Gourdon, P.; Liu, X.-Y.; Skjørringe, T.; Morth, J. P.; Møller, L. B.; Pedersen, B. P.; Nissen, P., Crystal structure of a copper-transporting PIB-type ATPase. *Nature* **2011,** *475* (7354), 59-64.
18. Notredame, C.; Higgins, D. G.; Heringa, J., T-Coffee: A novel method for fast and accurate multiple sequence alignment. *Journal of molecular biology* **2000,** *302* (1), 205-217.
19. Pettersen, E. F.; Goddard, T. D.; Huang, C. C.; Couch, G. S.; Greenblatt, D. M.; Meng, E. C.; Ferrin, T. E., UCSF Chimera—a visualization system for exploratory research and analysis. *Journal of computational chemistry* **2004,** *25* (13), 1605-1612.
20. Ozenne, V.; Bauer, F.; Salmon, L.; Huang, J.-r.; Jensen, M. R.; Segard, S.; Bernadó, P.; Charavay, C.; Blackledge, M., Flexible-meccano: a tool for the generation of explicit ensemble descriptions of intrinsically disordered proteins and their associated experimental observables. *Bioinformatics* **2012,** *28* (11), 1463-1470.
21. Estrada, J.; Bernadó, P.; Blackledge, M.; Sancho, J., ProtSA: a web application for calculating sequence specific protein solvent accessibilities in the unfolded ensemble. *BMC bioinformatics* **2009,** *10* (1), 104.
22. Humphrey, W.; Dalke, A.; Schulten, K., VMD: visual molecular dynamics. *Journal of molecular graphics* **1996,** *14* (1), 33-38.
